# Fluorescent markers rhodamine B and uranine for *Anopheles gambiae* adults and matings

**DOI:** 10.1186/s12936-020-03306-5

**Published:** 2020-07-06

**Authors:** Erica I. Aviles, Rachel D. Rotenberry, C. Mathilda Collins, Ellen. M. Dotson, Mark Q. Benedict

**Affiliations:** 1 Centers for Disease Control and Prevention, 1600 Clifton Road, Atlanta, GA 30329 USA; 2 Centre for Environmental Policy, Imperial College London, 16-18 Princes Gardens, London, SW7 1NE UK

**Keywords:** Mosquito, Marker, Fluorescent dye, Mating, Seminal fluid, Insemination, Auto-fluorescence

## Abstract

**Background:**

Marking mosquitoes is vital for mark-release-recapture and many laboratory studies, but their small size precludes the use of methods that are available for larger animals such as unique identifier tags and radio devices. Fluorescent dust is the most commonly used method to distinguish released individuals from the wild population. Numerous colours and combinations can be used, however, dust sometimes affects longevity and behaviour so alternatives that do not have these effects would contribute substantially. Rhodamine B has previously been demonstrated to be useful for marking adult *Aedes aegypti* males when added to the sugar meal. Unlike dust, this also marked the seminal fluid making it possible to detect matings by marked males in the spermatheca of females. Here, marking of *Anopheles gambiae* sensu stricto with rhodamine B and uranine was performed to estimate their potential contribution.

**Methods:**

Two fluorescent markers, rhodamine B and uranine, were dissolved in sugar water and fed to adult *An. gambiae*. Concentrations that are useful for marking individuals and seminal fluid were determined. The effects on adult longevity, the durability of the marking and detection of the marker in mated females was determined. Male mating competitiveness was also evaluated.

**Results:**

Rhodamine B marking in adults is detectable for at least 3 weeks, however uranine marking declines with time and at low doses can be confused with auto-fluorescence. Both can be used for marking seminal fluid which can be detected in females mated by marked males, but, again, at low concentrations uranine-marking is more easily confused with the natural fluorescence of seminal fluid. Neither dye affected mating competitiveness.

**Conclusions:**

Both markers tested could be useful for field and laboratory studies. Their use has substantial potential to contribute to a greater understanding of the bio-ecology of this important malaria vector. Rhodamine B has the advantage that it appears to be permanent and is less easily confused with auto-fluorescence. The primary limitation of both methods is that sugar feeding is necessary for marking and adults must be held for at least 2 nights to ensure all individuals are marked whereas dusts provide immediate and thorough marking.

## Background

Determining the characteristics of wild populations of mosquitoes such as population size, survival, dispersal and mating is challenging due to their small size, nocturnal habits and short life-spans. Some life-history traits can be determined in the laboratory, but the similarity of the parameter values to those observed in the field is often doubtful because the artificial environments do not reflect the stresses of existence in natural environments.

One technique to determine some of these characteristics, mark-release-recapture (MRR), consists of releasing marked mosquitoes into wild populations and observing them in subsequent collections [1], however the number of marking methods is limited. Dyes were one of the earliest methods, in one case sprayed as an aqueous mist on caged adults [2], which the author claimed was easily detected. Radioisotope methods that were once used for marking adults [3] and matings [4] have fallen out of use, presumably for safety reasons. The rare earth mineral rubidium has been used to mark individuals and eggs [5] but its use is limited by the detection methods which include mass spectroscopy and X-ray fluorescence [6]. Stable isotopes have also been used to detect matings by marked males but similarly [7], detection requires mass spectroscopy. Paint marking patterns [1] have been applied in unique patterns and colours for individuals in at least one case [8] and can reportedly be performed quickly by skilled staff but do not allow detection of matings.

By far, after Day-Glo™ pigment powders became widely available, the most commonly used method employs various types of this easily detected fluorescent dust [9]. Dusts can be applied quickly to adults and all individuals in a single cage can be marked rapidly and detected directly either using white light or, more sensitively, far blue or near-ultraviolet (UV) light. Several contemporary alternative indirect methods for studying life history characteristics described in the context of mark-release-recapture (MRR) have been expanded upon [10], and most of the methods have been described in detail [11].

Building upon the work of Welch et al. [12], the usefulness of rhodamine B for marking *Aedes aegypti* has been demonstrated [13]. Of particular note is that this red dye could be detected in seminal fluid that was transferred during mating. Given the apparent low toxicity and absence of an effect on mating competitiveness in *Ae.* *aegypti*, its utility for similar purposes in *Anopheles gambiae* was determined. The current interest in field studies of *An. gambiae* is largely related to genetic control [14, 15]. This is an application for which a new marker that could easily detect matings would be extremely useful, but we anticipated that the more delicate *An. gambiae* might respond differently to marking than *Ae. aegypti* and that the methods might need to be modified.

In order to develop another marker with a different colour, a related dye, uranine which has yellow-green fluorescence, was also assessed. The results demonstrate that the different characteristics of the dyes expand the options for *An. gambiae* marking. While under certain circumstances releases of females can safely be performed [10], the primary focus in these experiments was on marking males.

## Methods

All mosquitoes used were *An. gambiae* ‘G3’ strain (MR4 number MRA-112, BEI Resources, Manassas, VA). Mosquitoes were cultured using the Damiens larval diet [16] fed according to a standard operating procedure [17]. The light cycle was 12:12 light:dark with 30 m sunrise and sunset during the 12 h light period. The observed air temperature and percent relative humidity (%RH) during the period of the observations were 27.4 **°**C and 80.4% RH (std dev 0.78 and 7.0, respectively). Adults were placed in aluminum cages [18] covered with tubular gauze (TG12 Stockinette, Bandages Plus, Doral FL) and allowed to feed on sugar water containing marker for 3 nights except during preliminary observations as noted below.

Two candidate fluorescent markers were tested, rhodamine B (Product no. R6626-25G, Sigma-Aldrich, St. Louis MO, hereafter called simply ‘rhodamine’) and uranine (Product no. 108462, Millipore-Sigma, Burlington MA, USA). These were dissolved in 10% w/v food-grade sucrose containing 0.1% w/v methylparaben (ME163, Spectrum, New Brunswick NJ, USA [19]) which will be described simply as ‘sugar water’. Dyes were prepared at various concentrations (all  % w/v) and supplied to mosquitoes on cotton balls soaked with 20 ml of each solution in 59 ml polypropylene cups (DuraHome Product no. X001QYETQV, Amazon, Seattle WA USA). Adults were immobilized by chilling on ice for examination and sorting. Adult marking was detected with an SZX-ZB7 microscope (Olympus Waltham, MA) using an XCite 120Q illuminator (Model no. C182979, Excelitas, Waltham, MA) using filter cubes for detection of DsRed, GFP or both simultaneously (Nos. 49004, 49020 and 59004, respectively, Chroma, Marlborough, MA USA). Adults were observed at magnifications from 8 to 56×.

Statistical analyses were performed using R 3.5.1 “Feather Spray” [20]. Where the data were a single value, or a proportion derived from numbers in each case (dead/alive, mated/not mated) per replicate, generalized linear models (GLM) were used and the contribution of fixed effects was assessed by deletion tests using F or χ^2^ as appropriate to the dispersion pattern seen in the data.

Mixed effects models, which account for the pseudoreplication inherent with repeat measures from within experimental units/replicates, were used to analyse the effects of dye concentration on longevity. These used the ‘nlme’ package [21]. Further details are provided below.

### Survival of males in the absence of sugar water

Because vitality and marking via sugar water requires males to consume dyed sugar water, we determined how long males could live without sugar water. This would indicate the period of time that might be necessary for feeding dye that would ensure all living males had consumed the dye. Male pupae (n = 48) were placed in a cage that contained no sugar water. Using the light schedule of this insectary, adults generally emerge shortly after the light period on the evening from pupae that form before approximately 10:00 a.m., therefore, males on the following day were considered to be 1 d old. The pupa cup was removed the following day (day 1) to ensure the males could not drink water in the pupa cup.

### Acute toxicity

To determine the range of dye concentrations within which marking was detectable but which did not cause obvious acute mortality, tests were performed with concentrations we expected to be on the low and high ends of a useful range. The previous report [13] described marking *Ae.* *aegypti* adults using rhodamine at concentrations ranging from 0.1 to 0.8%. Based on those values, we performed preliminary observations on rhodamine acute toxicity and marking at 0.1, 0.2, and 0.8%. Three cages of 20 males each were set up at a concentration of 0.8% and two at 0.1 and 0.2%, mosquitoes were allowed to feed for 3 nights and mortality recorded on the fourth day.

For uranine, three cages of 25 males were set up at each of 0.1 0.2, 0.4 or 0.8% concentration in sugar water. As with rhodamine, mortality was recorded on the fourth day. A binomial generalized linear model (GLM) was used to estimate any pattern in variation of mortality between concentrations.

### Detecting marker when fed at different concentrations

After observing very distinct marking at doses ≥ 0.2% rhodamine, three lower concentrations were added, 0.1%, 0.05% and 0.025%.

### Durability of marking

To determine how long the markers were detectable in adults, three cages of mosquitoes that had been marked for 3 nights (50 females and 50 males) with 0.1 and 0.2% rhodamine or with 0.1, 0.2, 0.4 or 0.8% uranine were held in separate cages along with three unmarked control cages for each dose. Samples were removed at weekly intervals for 3 weeks and the number in which the marker was detectable was determined. Ten of each sex per cage were examined at the end of weeks 1 and 2. In some cases there were not 10 of each sex remaining alive by week 3.

For each sample, the numbers marked and unmarked were bound together in a weighted binomial response variable. A quasi-binomial GLM was used to explore any pattern in variation of the proportion of mosquitoes detectable as marked as a function of the sex of the mosquitoes, the dye concentrations, time since marking (in weeks—fit as a factor rather than a linear term) and their two-way interaction terms on the proportion identified as marked was estimated. The cage identity was also fit to the model to account for any cage effects. Effects were assessed by deletion testing of factors, interactions and factor-level simplifications.

### Detection of mating by marked males

Cups of approximately 100 male pupae were placed in cages and the resulting adults were exposed to 0.05, 0.1, or 0.2% rhodamine or 0.1, 0.2 or 0.4% uranine in sugar water for 3 nights. On the following day, the adults were chilled and examined to ensure the marker was visible in all individuals. Thirty virgin females were added to each cage. Adults were provided an opportunity to mate for 4–7 nights. Matings were observed by examining spermathecae dissected in Fluoroshield with DAPI (F6057—20 ml, Sigma-Aldrich, St. Louis, MO) under a coverslip. Spermathecae were first examined without squashing, and after examination they were squashed using light pressure and were observed sequentially for sperm and fluorescence on an Olympus BX60 at 40–200× magnification and then on an Evos Fl (Thermo Fisher, Grand Island, NY) at 10–20× using the RFP filter cube for rhodamine (AMEP 4625, Thermo Fisher) or the GFP filters for uranine (AMEP 4651, Thermo Fisher).

### Spermatheca marking durability

To determine whether spermatheca marking persisted, spermathecae were observed at intervals after females were given the opportunity to mate and males removed to determine whether fluorescence remained visible. Males that were confirmed as marked after having been fed either 0.8% uranine or 0.2% rhodamine were mated separately to pools of approximately 100 G3 females for 4 (rhodamine) or 3 (uranine) days. Males were then removed and spermathecae dissected at intervals.

### Competitive matings

Competitive matings were performed by combining 20 confirmed rhodamine-marked and 20 unmarked males with 40 virgin G3 females. Males had been maintained on either sugar water or 0.1 or 0.2% rhodamine sugar water for 2 nights or 0.1, 0.2 or 0.4% uranine. Spermathecae were observed as described above, usually within 2 days of the end of the mating period.

The data consisted of the numbers mated and unmated, and, of those mated, by which male type (marked or not marked) in each replicate. These were bound together as binomial response variables and a quasi-binomial GLM was used to assess effects of marker and dose on these measures (weighted proportion of females mating or mating with fluorescent males). This method allowed for the over-dispersion that was observed in the data (residual deviance was observed to be > residual df). Because the design was not fully orthogonal (rhodamine had fewer dose levels than uranine), dose was treated as a factor with three levels rather than as a linear measure.

### Longevity

For longevity studies, only adult males and females in which the marker had been observed were used for tests. Adults were allowed to feed on 0.05 or 0.1% rhodamine in sugar water for 3 nights, anesthetized on ice and examined as described above. Three cages of 30 confirmed marked individuals at each concentration or of unmarked males and females were set up and adults were provided sugar water. Mortality was observed daily except some Saturdays until all adults were dead.

The data arising from this experiment are a daily-interval time series for each cage. Because different doses were considered appropriate based on the acute lethality for rhodamine and uranine, their data were considered separately. To allow for the temporal pseudo-replication arising from repeated measurement of sequentially-linked cohorts (cage replicates), mixed effects models were used to identify whether there was a significant effect on the proportion surviving over time as a function of mosquito sex, the dose of marker received and whether the other sex they were caged with was itself dosed with marker. The interactions of sex and dose were also estimated. Random effects were used represent the pseudo-replication of within-cage trajectories. The survival was compared over the period of the experiment (42 days maximum) and assessment of the main effects and their interactions was by model simplification using L-Ratio tests at p < 0.01 to avoid over-interpretation, with Akaike Information Comparisons (AIC) to evaluate model fit.

## Results

### Survival in the absence of sugar water

All adults successfully enclosed and were alive in the cage on day 1. On day 2, 12 males were dead and on day 3 the remaining 36 males had died. A minimum of 2 nights of feeding is necessary to ensure all males have imbibed the dyed sugar water or that they would die under the experimental environmental conditions. As a precaution, males were routinely marked for 3 nights.

### Acute dye toxicity

#### Rhodamine acute toxicity

Of the three cages of males exposed to 0.8% rhodamine, after 3 nights of exposure, all males in two of the cages had died and this dose was not pursued further. In observations of the three cages of 20 males treated at 0.2%, 11, 4, and 5 males average mortality = 33%) were dead after 2 nights of exposure and we continued observations at this and lower doses.

#### Uranine acute toxicity

After 4 days, little mortality was observed at any concentration (n = 0, 3, 3, 2/50 at 0.1, 0.2, 0.4 or 0.8% uranine, respectively) and there was no indication that mortality varied with concentration (F = 1.65, d.f. = 8,11, p = 0.18).

### Durability of marking

**Fig. 1:**
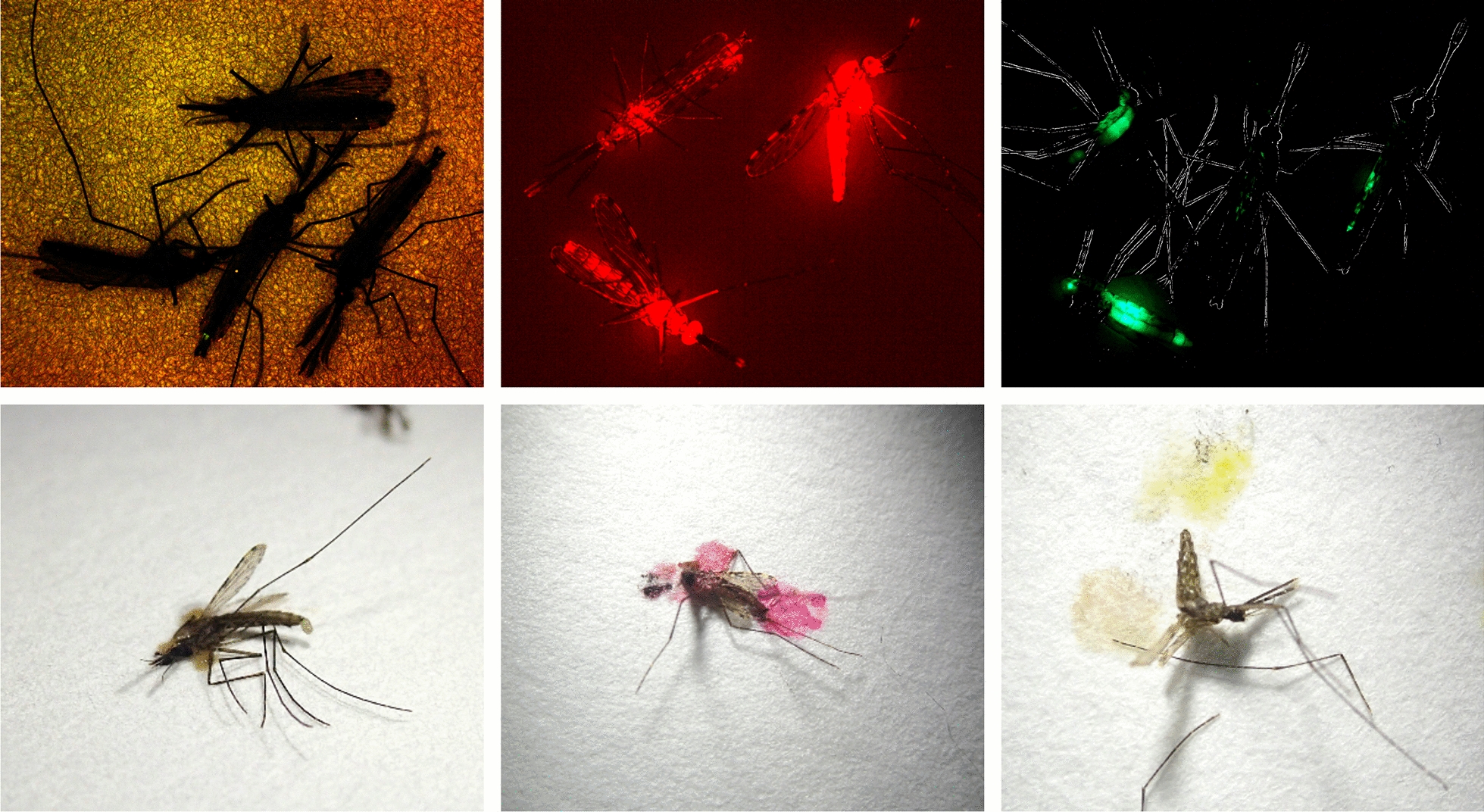
Appearance of marker. *Anopheles gambiae* adults that were unmarked (left column), marked with 0.1% rhodamine (center) or 0.2% uranine (right) were examined by fluorescence microscopy (upper row) or squashed on filter paper. Due to the low intensity of the uranine marking, the outlines of the adults were overlaid over the fluorescence image. The image of the unmarked adults was greatly overexposed relative to the marked images to make them visible

When adults were marked with 0.1 and 0.2% rhodamine, marker was detected every week for 3 weeks after marking in all individuals that were alive (0.1%, 93 females, males 79; 0.2%, 89 females, 76 males). In the 0.1% treatment cages, 33 females and 19 males remained alive at week 3. By the final week of the 0.2% treatment cages, 29 females and 16 males were alive. No marker was identified among the living rhodamine controls (69 females, 53 males). Because all living treated adults remained marked, no statistical analysis was warranted (Fig. 1).

**Fig. 2:**
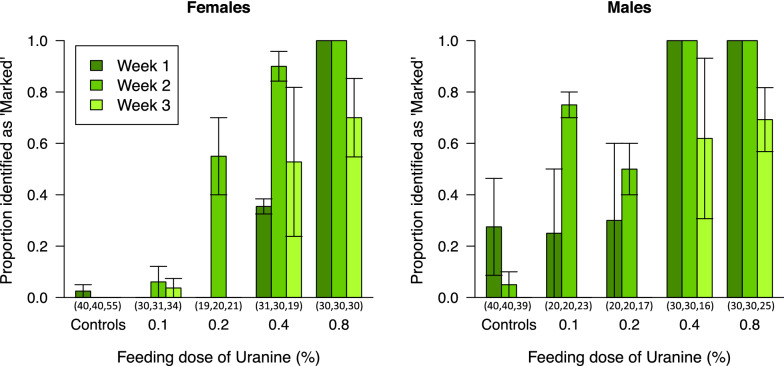
Durability of uranine marking. The proportion of female and male *An.* *gambiae* (mean of three treatment and four control cage samples ± SEM) identified as ‘marked’ by feeding with increasing concentrations of uranine and evaluated for 3 weeks post-marking. Doses of 0.2% and below were not distinguishable from natural auto-fluorescence (p > 0.05), males were more rapidly identified as marked than females (p < 0.001), higher doses are more rapidly identified and last longer (p < 0.05). The number analysed for each of the 3 weeks is shown in parentheses below the X axis

Uranine produces a yellow-green colour that is similar to natural autofluorescence and, at low treatment doses, these can be difficult or impossible to distinguish (Fig. 1). The proportion identified as uranine-marked in control cages did not vary among the different trials and all control cages were considered as one level (F = 0.03, d.f. = 55,61, p = 0.99) (Fig. 2). The level of false positives in control treatments led us to ask at what dose would treatment effects be clearly distinguishable from these. In both sexes, the proportion identified as marked did not differ from controls in either dose levels 0.1 or 0.2% (F = 0.88, d.f. = 61,63, p = 0.42 and F = 2.28, d.f. = 67,68, p = 0.14 respectively). These results should be interpreted against a background of false positives that were detected: 1 of 135 unmarked females (0.07%) and 13 of 119 unmarked males (11%) were scored as marked. This error would be most influential if adults had been marked with low doses and were analysed 2 or 3 weeks after release. In this regard, rhodamine is clearly the superior marker. The uranine results also present contradictions that we attribute to the judgment that must be exercised when classifying individuals as marked. For example, at 0.1% marking, the number of males classified as marked actually increased in week 2 compared to week 1.

A higher proportion of male mosquitoes were identified as ‘marked’ (F = 14.45, d.f. 66,67, p = 0.0003), but male and female mosquitoes responded similarly to the dose received and through time (sex:week F = 2.72, d.f. = 65,66, p = 0.10; sex:dose F = 1.22, d.f. = 66,68, p = 0.30). There was, however, an interaction between dose and week (F = 4.48, d.f. = 68,70, p = 0.015); at low doses, marking can both take time to appear and the proportion identifiable as marked declines more rapidly.

At no dose was the marking visible in all individuals for the full 3 weeks in either sex though all mosquitoes marked at 0.8% were detectable after 1 and 2 weeks. All males marked at 0.4% were detectable for 2 weeks. One should though bear in mind that false positive males, as noted in the in the control group, may also contribute to the totals scored as marked in the other groups.

### Detection of mating by marked males

Results of the detection of mating are shown in Table 1. Only one test at the dose rate of 0.05% rhodamine was conducted after it was determined that the marker could not be reliably detected in males when males were fed at this rate. In spite of this, the marker could be detected in all matings from males marked with 0.05% rhodamine; though the number was small, as a mating marker it appears useful at concentrations that are not high enough for detecting the marker in the male body. At all doses of rhodamine, matings with the marker could be easily detected in almost all females. At the lowest dose of uranine (0.2%), the marker could be detected in the spermathecae although the marker could not be reliably detected in the adult males themselves.

**Table 1:** Sperm in spermathecae (n) with (Fluor.) and without (Neg.) fluorescent spermathecae

Marker dose %	Exps. (n)	With Sperm		No sperm	
		Fluor.	Neg.	Fluor.	Neg.
Rhodamine					
0.05	1	7	0	0	10
0.1	3	55	0	0	20
0.2	3	35	0	0	47
Total	7	97	0	0	77
Uranine					
0.1	2	6	0	0	12
0.2	1	7	3	0	5
0.4	3	60	0	0	20
0.8	3	59	0	0	27
Total	6	73	3	0	37

### Spermatheca marking durability

Spermathecae that were positive for sperm were all positive for rhodamine at 3, 11, 17 and 24 days after mating (positive/negative, 8/14, 6/4, 8/2, 1/0). In the other experiment using uranine marking, spermathecae that were positive for sperm were all positive for uranine when dissected and observed at 4, 11, 18 and 25 days (positive/negative, 7/15, 9/1, 9/1, 8/2, respectively).

### Competitive matings

**Fig. 3:**
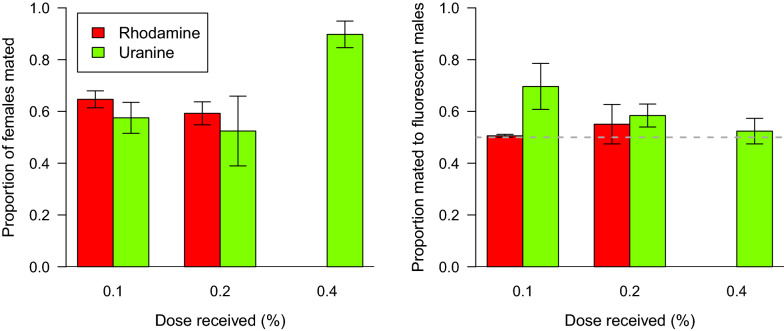
Competitive mating. The mean proportion (± SEM) of female mosquitoes mated overall (left) and, (right) the proportion (± SEM) of those mating with fluorescent males as a function of the marker used and dose received. The dotted line represents the assumption of equal proportions of females mated by either type of male. Slightly more females were mated by treated males than by control males (p < 0.05), neither marker, nor dose affected this statistically (p > 0.05)

A slightly greater proportion of females were mated by marked males than by unmarked males (mean = 0.57, 95% CI = 0.51–0.63, χ^2^ = 23.93, d.f. = 14, p = 0.047, Fig. 3).

The treatments, however, did not affect this proportion. The effect of dosage did not depend on the marker used (F = 2.79, d.f. = 10,11, p = 0.13). The dose received itself did not affect choice of male type (F = 1.97, d.f. = 11,13, p = 0.19) nor did the marker used (F = 1.18, d.f. = 13,14, p = 0.30).

### Longevity

In neither marker trial, did the marker status of the accompanying mosquitoes in the cage affect survival patterns (uranine: L. ratio = 0.48, d.f. = 9,10, p = 0.49; rhodamine: L. ratio = 0.10, d.f. = 9,10, p = 0.76). With uranine, the two mosquito sexes responded differently to dosage; males generally had shorter lives than females, but were not as sensitive to increasing dye concentrations as were females (Table 2, L. ratio = 58.38, d.f. = 8,9, p < 0.001). With rhodamine, longevity in control males was also shorter than for control females and, although the range of doses was low, longevity also declined less precipitously with increasing dye concentration than seen in females (Table 2, L. ratio = 14.37, d.f. = 8,9, p < 0.001).

**Table 2:** Adult longevity of dye-treated adults

Median day of 50% mortality	Concentration of fluorescent dye (%)				
	0.0	0.1	0.2	0.4	0.8
Uranine					
Female	28	26	24	24	21
Male	21	20	24	20	19
Rhodamine					
Female	27	27	21	ND^a^	ND
Male	19	9	8	ND	ND

^a^Not done

## Discussion

### Detecting marker when fed at different concentrations

Recommended concentrations for *An. gambiae* of rhodamine are 0.1 and 0.2% and for uranine 0.2, 0.4 and 0.8%. At these concentrations, mortality is not substantial and rhodamine can be detected permanently in adults and easily in the spermathecae of females mated to marked males. These results do not represent experience with all strains so researchers should consider testing the markers on the specific strains that they plan to use. We have received one report (L Facchinelli, pers. comm.) that the “Banfora” (*Anopheles coluzzii*) strain suffers significant mortality after 72 h of 0.2% rhodamine exposure in their hands whereas the “Kisumu” (*An. gambiae*) strain did not. Therefore, those who are considering the use of either rhodamine or uranine are advised to test for possible toxicity effects before applications are developed.

Insects can be marked individually or *en masse* [22]. The most common marker for mosquitoes by far is DayGlo type fluorescent dusts [9]. Among the mass-marking methods, in comparison with rhodamine and uranine, dusts have both advantages and disadvantages. Perhaps the greatest advantage of dust is one’s ability to apply it to a large number of caged mosquitoes simultaneously and quickly. It is not necessary to hold them until they have fed on the marker which means the adults must be 3 days old before marking is ensured. On the other hand, dusts can rub off and if the amount is not great, it can be difficult to see. It provides no marking for mating studies. Dust can also be transferred between individuals in traps and via contamination in collection devices whereas rhodamine and uranine cannot. After feeding on dyes, adult urine was stained with dye, so it is possible that droplets could fall on other mosquitoes. These well-defined droplets would not easily be confused with the diffuse internal marking but it is possible. The effect of marking on adult survival should be also be considered. While there were no strong effects detected at the recommended doses, it is possible this will be observed in some strains or in the field. Feeding dye requires little judgment or experience whereas different methods for applying dust can affect the survival of adults [23]. The external application of a fluorescent dye in a water mist has been reported [23] and compared with dust and controls, but was considered inferior for detection and survival whereas no effect of dust was observed.

In our hands, rhodamine adult marking at 0.1 and 0.2% is permanent for at least 3 weeks. In contrast, uranine gradually becomes undetectable and cannot be distinguished reliably from autofluorescence at the lowest doses and thus must be used at concentrations of at least 0.4% for male marking up to 2 weeks and at 0.8% for females. While this might be considered a disadvantage for most uses, in some cases, the persistence of previously marked adults interferes with subsequent releases using the same marker in which case this decline in detectability could be advantageous.

Both uranine and rhodamine are useful mating markers. Rhodamine is more specific and sensitive than uranine as the male accessory gland fluid has weak yellow-green fluorescence that is easily confused with low-dose uranine marking. Therefore, at low feeding concentrations, it requires practice to distinguish normal fluorescence from uranine. In many cases, sperm were not initially detected in the spermatheca, but after observing rhodamine fluorescence, re-examined the spermatheca under white light and located them. Therefore, rhodamine is possibly a more sensitive marker for mating than observing sperm directly, but we do not know whether seminal fluid is sometimes transferred with no, or few, sperm. Rhodamine appears to be concentrated in the male accessory gland (pers. comm. P. Bascuñán), possibly explaining why it can be detected more easily than sperm.

The limitations and potential applications of these markers and applications for laboratory studies has been described and it is expected that these can be replicated by other researchers. However, it is yet to be seen to what extent the additional stresses of natural environments might affect their utility. More extreme humidity and temperatures, interactions with hosts and expanded flight ranges can all potentially affect the persistence of the markers. Increased metabolism and higher temperatures particularly may affect the persistence of the markers.

No effect of either marker on male mating competition was detected. This is similar to the observations of Johnson [13] and indicates real promise for this marking purpose and these dyes in *An. gambiae* sensu lato. When uranine is used at low concentrations however, interpreting outcomes may be difficult. While no difference was identified, it is possible that some non-marked uranine male matings were mistaken for uranine marked male matings, particularly at the lowest dose. Moreover, multiple matings, which are known to occur [24], by both a marked and unmarked male would likely be classified as only being due to a marked male.

## Conclusions

Both rhodamine and uranine fed at appropriate concentrations for each dye are promising and useful for marking of *An. gambiae* adults and for detecting matings. Their use in both laboratory and field studies of these mosquitoes has substantial potential to contribute to a greater understanding of the bio-ecology of this important malaria vector. Researchers who are considering either of these markers should confirm that the chosen marker does not affect the life-history traits in their study strains or local wildtype mosquitoes that are most important to the outcomes of their experiments.

## Data Availability

The data are available only upon request from the authors.

## Abbreviations

GLM: General linear model
GFP: Green fluorescent protein
RH: Relative humidity
Stdev: Standard deviation
UV: Ultraviolet
